# FibroScan-AST Score Predicts 30-Day Mortality or Need for Mechanical Ventilation among Patients Hospitalized with COVID-19

**DOI:** 10.3390/jcm10194355

**Published:** 2021-09-24

**Authors:** Marko Zelenika, Marko Lucijanic, Tomislav Bokun, Tonci Bozin, Mislav Barisic Jaman, Ida Tjesic Drinkovic, Frane Pastrovic, Anita Madir, Ivica Luksic, Nevenka Piskac Zivkovic, Kresimir Luetic, Zeljko Krznaric, Rajko Ostojic, Tajana Filipec Kanizaj, Ivan Bogadi, Lucija Virovic Jukic, Michal Kukla, Ivica Grgurevic

**Affiliations:** 1 Department of Gastroenterology, Hepatology and Clinical Nutrition, University Hospital Dubrava, 10000 Zagreb, Croatia; marko.zelenika@gmail.com (M.Z.); tbokun@gmail.com (T.B.); tbozin@gmail.com (T.B.); mislav.barisic.jaman@gmail.com (M.B.J.); ida.tjesicdrinkovic@gmail.com (I.T.D.); fpastrovic@gmail.com (F.P.); 2 Department of Hematology, University Hospital Dubrava, 10000 Zagreb, Croatia; markolucijanic@yahoo.com; 3 School of Medicine, University of Zagreb, 10000 Zagreb, Croatia; anita.madir@gmail.com (A.M.); luksic.ivica@gmail.com (I.L.); zeljko.krznaric1@zg.t-com.hr (Z.K.); rajko.ostojic@gmail.com (R.O.); tajana.filipec@gmail.com (T.F.K.); lucija.jukic@gmail.com (L.V.J.); 4 Faculty of Pharmacy and Biochemistry, University of Zagreb, 10000 Zagreb, Croatia; 5 Department of Maxillofacial Surgery, University Hospital Dubrava, 10000 Zagreb, Croatia; 6 Department of Pulmology, University Hospital Dubrava, 10000 Zagreb, Croatia; npiskac@gmail.com; 7 Department of Gastroenterology and Hepatology, University Hospital Sveti Duh, 10000 Zagreb, Croatia; kresimir.luetic@hlk.hr; 8 Department of Gastroenterology and Hepatology, University Hospital Centre Zagreb, 10000 Zagreb, Croatia; 9 Department of Gastroenterology and Hepatology, University Hospital Merkur, 10000 Zagreb, Croatia; ivanbogadi08@gmail.com; 10 Department of Gastroenterology and Hepatology, University Hospital Sestre Milosrdnice, 10000 Zagreb, Croatia; 11 Department of Internal Medicine and Geriatrics, Faculty of Medicine, Jagiellonian University Medical College, 30688 Cracow, Poland; michal.kukla@uj.edu.pl

**Keywords:** COVID-19, liver, non-alcoholic steatohepatitis, transient elastography, mortality

## Abstract

Background: Liver involvement in Coronavirus disease 2019 (COVID-19) has been recognised. We aimed to investigate the correlation of non-invasive surrogates of liver steatosis, fibrosis and inflammation using transient elastography (TE) and FibroScan-AST (FAST) score with (a) clinical severity and (b) 30-day composite outcome of mechanical ventilation (MV) or death among patients hospitalized due to COVID-19. Method: Patients with non-critical COVID-19 at admission were included. Liver stiffness measurement (LSM) and controlled attenuation parameter (CAP) were assessed by TE. Clinical severity of COVID-19 was assessed by 4C Mortality Score (4CMS) and need for high-flow nasal cannula (HFNC) oxygen supplementation. Results: 217 patients were included (66.5% males, median age 65 years, 4.6% with history of chronic liver disease). Twenty-four (11.1%) patients met the 30-day composite outcome. Median LSM, CAP and FAST score were 5.2 kPa, 274 dB/m and 0.31, respectively, and neither was associated with clinical severity of COVID-19 at admission. In multivariate analysis FAST > 0.36 (OR 3.19, *p* = 0.036), 4CMS (OR 1.68, *p* = 0.002) and HFNC (OR 7.03, *p* = 0.001) were independent predictors of adverse composite outcome. Conclusion: Whereas LSM and CAP failed to show correlation with COVID-19 severity and outcomes, FAST score was an independent risk factor for 30-day mortality or need for MV.

## 1. Introduction

Coronavirus disease 2019 (COVID-19) is respiratory disease with multisystem involvement and is responsible for a worldwide pandemic [1]. Reported overall mortality of hospitalized patients is approximately 20%, with considerable variability due to age, comorbidity, level of care and thresholds for hospitalization [2,3,4,5,6]. Risk factors for severe clinical course include older age, male sex, comorbidity, arterial hypertension, diabetes mellitus and obesity [2,3,4,5,6,7].

Non-alcoholic fatty liver disease (NAFLD) is considered the liver manifestation of the metabolic syndrome that includes concomitant presence of obesity, hypertension, diabetes mellitus and dyslipidaemia. NAFLD affects around 25% of adults worldwide, with even greater prevalence among obese individuals (>50%) and those having additional metabolic conditions [8,9]. In line with these facts, the presence of fatty liver might be assumed as the risk factor for more severe forms of COVID-19 and adverse outcomes [10]. In addition, increased hepatic expression of angiotensin-converting enzyme II (ACE2) receptors, as the gate for viral entry in the cells has been demonstrated in patients with non-alcoholic steatohepatitis (NASH) [11]. Several studies have investigated liver involvement in COVID-19, demonstrating worse clinical course of patients with elevated liver functional tests (LFTs) and the presence of cirrhosis [12,13,14]. On the other hand, patients with non-cirrhotic chronic liver disease (CLD) seem to have comparable survival to patients without CLD. However, more specific data on the prevalence, severity and prognostic impact of liver steatosis, fibrosis and non-alcoholic steatohepatitis (NASH), on the course of COVID-19 are scarce with conflicting results published [15,16,17,18]. In fact, there are no specific data considering the impact of NASH, as the inflammatory phenotype of NAFLD, because liver biopsy as the gold standard method to diagnose NASH is rarely performed in the setting of COVID-19. Recently, a non-invasive tool has been developed, called the Fibroscan-AST (FAST) score. This score combines the results of liver stiffness measurement (LSM), steatosis assessment by controlled attenuation parameter (CAP) and aspartate aminotransferase (AST) and has been developed to identify patients with NASH, elevated NAFLD activity score (NAS ≥ 4) and significant fibrosis (fibrosis stage F ≥ 2) amongst those with NAFLD [19]. In theory, patients who already have liver inflammation due to NASH (as opposed to those with bland steatosis without NASH, and those with a healthy liver) might react with more profound inflammatory response upon contracting COVID-19 and thus have worse clinical outcomes.

In the present study we aimed to investigate the correlation of non-invasive surrogates of liver steatosis, fibrosis and inflammation using transient elastography (TE) and FAST score with (a) severity of clinical presentation and (b) 30-day composite outcome of mechanical ventilation (MV) or death among patients hospitalized with non-critical form of COVID-19 at admission.

## 2. Patients and Methods

### 2.1. Patients

This study was performed in the university hospital that was completely re-purposed to serve exclusively as the main regional centre of care for COVID-19 patients during the 2020/2021 pandemic. Those considered for inclusion were non-critically-ill patients admitted to general wards, capable of giving informed consent, over a 3-month period (16 January to 17 April 2021). All patients had positive nasopharyngeal swab for severe acute respiratory syndrome coronavirus 2 (SARS-CoV2) by polymerase chain reaction (PCR) and were admitted following initial examination in the emergency department. Standard work-up in emergency department included blood biochemistry, electrocardiogram, peripheral blood oxygen saturation and chest X-ray. The clinical severity of COVID-19 was initially assessed by using Modified Early Warning Score (MEWS) for each patient [20]. Patients with non-critical form of the disease (MEWS < 5) were admitted to the general ward and those with MEWS ≥ 5 were admitted to intensive care unit (ICU). Even if deemed non-critical by MEWS, all patients required oxygen supplementation therapy, and in order to further stratify them, the 4C Mortality Score (4CMS) was calculated for each patient who was hospitalised [21].

Data about the presence of pre-existing chronic liver disease (CLD), including the presence of cirrhosis and previous decompensation, as well as about the alcohol consumption were collected by history taking from each patient and supported by previous medical records. Harmful alcohol intake was considered in patients consuming >30 g/day (males) and >20 g/day (females).

Upon admission to the ward patients were treated by oxygen supplementation by binasal catheters, masks, or high-flow nasal cannula (HFNC) as needed. Other treatment such as dexamethasone or equivalent methylprednisolone dose, remdesivir and low-molecular-weight heparin were instituted according to recommendations by national guidelines [22]. Other therapy was commenced at the discretion of the attending physician, including antibiotics, as well as other medications to treat acute complications and the patients’ underlying chronic conditions.

Liver stiffness measurements (LSM) and steatosis assessment by controlled attenuation parameter (CAP) were performed within 72 h from the admission in patients meeting the inclusion criteria: age > 18 years, signed informed consent, non-critical form of COVID-19 at admission (MEWS < 5), and absence of conditions affecting liver stiffness measurement (LSM) (ALT > 5xULN, congestive liver disease, extrahepatic biliary obstruction, infiltrative liver neoplasms) [23].

### 2.2. Methods

#### 2.2.1. Transient Elastography

Transient elastography (TE) was used to perform LSM and CAP measurements by using Fibroscan device integrated within General Electric Logiq S8 XD Clear ultrasound platform. These examinations were performed in patients after overnight fasting, in the supine position with the right arm in the maximal abduction during the short (3–4 s) apnea period in the neutral breathing position, through the right intercostal approach as recommended by international guidelines [23]. The choice of using Fibroscan M or XL probe was made upon suggestion of automatic probe selection tool embedded within the Fibroscan device [24]. Ten valid LSM had to be performed with the interquartile range of LSM (IQR/Med) < 30%. CAP was automatically measured along with the LSM acquisitions. All examinations were performed by experienced operators (each had previously performed >500 TE examinations).

As the surrogate measure for the presence of significant fibrosis (F ≥ 2) in the analysed cohort LSM > 7 kPa was used, whereas LSM ≥ 10 kPa was considered representative for advanced fibrosis (F ≥ 3) [25,26]. Presence of liver steatosis (S > 0) was considered in patients with CAP > 274 dB/m [27].

#### 2.2.2. Laboratory Tests

Results of blood biochemistry (complete blood counts (CBC), urea, AST, alanine aminotransferase (ALT), gamma glutamyl transferase (GGT), alkaline phosphatase (ALP), bilirubin, prothrombin time (Quick,%), C-reactive protein (CRP)) were obtained no more than 48 h from the time of TE procedure and were recorded for the purpose of this study, except for the patients having HFNC oxygen supplementation in which case we limited this time period to 24 h because of their more unstable clinical course.

FAST score was calculated for each patient using the formula provided by Newsome PN et al.: FAST = (e^−1.65 + 1.07 × In(LSM) + 2.66 ∗ 10−8 × CAP3 − 63.3 × AST−1^)/(1 + e ^−1.65 + 1.07 × In(LSM) + 2·66 ∗ 10−8 × CAP3− 63.3 × AST−1^) [19]. Using the cut-offs of 0.35 and 0.67, FAST score had ≥90 sensitivity and ≥90% specificity, respectively to rule-out and rule-in the presence of NASH, elevated NAFLD activity score (NAS ≥ 4) and significant fibrosis (fibrosis stage F ≥ 2) amongst NAFLD patients in the original study.

As biochemical non-invasive scores for liver fibrosis have also been associated with the outcomes of COVID-19 patients in some reports [28,29], we calculated the following scores according to published formulae in order to compare their prognostic performance to TE:

AST to platelet ratio index (APRI), APRI = ((AST/ULN)/platelet count (×109/L)) × 100 [30];

Fibrosis-4 index (FIB-4), FIB4 = Age (years) × AST (IU/L)/platelet count (×109/L) × ALT (IU/L)1/2 [31];

NAFLD fibrosis score (NFS) = −1.675 + (0.037 ∗ age (years)) + (0.094 ∗ BMI (kg/m^2^)) + (1.13 ∗ Impaired fasting glucose/diabetes (yes = 1, no = 0)) + (0.99 ∗ AST/ALT ratio) − (0.013 ∗ platelet count (×109/L)) − (0.66 ∗ albumin (g/dL)) [32].

### 2.3. Statistical Analysis

Power analyses were based on assumption of 10% and 25% event rates in the subgroups of interest, type I error of 0.05 and 80% power, suggested that 200 patients had to be included to obtain a statistically significant result. Normality of distribution of numerical variables was tested using the Shapiro–Wilk test. Most of the analysed variables were non-normally distributed and as such all numerical variables are presented as median and interquartile range (IQR) and were compared between groups using the Mann–Whitney U test. Categorical variables are presented as frequencies and percentages and were compared between groups using the Χ^2^ test or the Fisher test where appropriate. Variables acquired by TE (LSM, CAP) and FAST score were compared to the clinically defined outcomes: (a) severity of COVID-19 clinical presentation as assessed by 4CMS or the need for HFNC oxygen supplementation and (b) 30-day mortality or need for MV. ROC curve analysis was used to establish optimal cut-off values of different elastographic measurements for prediction of 30-day mortality. Logistic regression was used to test independent contribution of particular variables to 30-day mortality prediction. *p*-values < 0.05 were considered statistically significant. All analyses were performed using the MedCalc statistical software version 20 (MedCalc Software Ltd., Ostend, Belgium).

## 3. Results

### 3.1. Patient Characteristics

Of 230 patients considered eligible, 217 patients with a non-critical form of COVID-19 at admission (MEWS < 5) were included in the study. In 13/230 patients, LSM was not possible due to obesity or dyspnoea. There were 144/217 (66.4%) males, median age was 65 years, IQR (55–70), median BMI was 28.3 kg/m^2^, IQR (25.4–31.5), 70/217 (32.3%) of patients had diabetes and 118/217 (54.4%)—arterial hypertension. Among the participants, 45 (21.1%), 89 (41.8%) and 79 (37.1%) patients had BMI < 25 kg/m^2^, 25–30 kg/m^2^ and >30 kg/m^2^, respectively. History of liver disease was present in 10/217 (4.6%) patients (4 with non-alcoholic fatty liver disease (NAFLD), 4 with alcoholic liver disease (ALD) including 1 with cirrhosis, and 2 patients with chronic hepatitis B). Bilirubin, AST, ALT, GGT and ALP were elevated in 11.5%, 55.3%, 42.4%, 44.2% and 6% patients, respectively. Median time from the initial COVID-19 symptoms to admission was 5 days IQR (1–9). All patients required oxygen supplementation, including 24/217 (11.1%) patients who required HFNC. Median 4C COVID-19 mortality score was 7, IQR (5–9) with 24/217 (11.1%) patients requiring ICU admission and mechanical ventilation (MV) and 22/217 (10.1%) patients dying within 30 days of admission. Patient characteristics and outcomes are summarized in Table 1.

### 3.2. Correlations of LSM and CAP with Demographic, Biochemical and Clinical Parameters

Median LSM was 5.2 kPa, with 41/217 (18.9%) patients presenting with LSM of >7 kPa and 12/217 (5.5%) patients with LSM ≥ 10 kPa. LSM was higher in males (5.3 vs. 4.9; *p* = 0.026), patients with history of chronic liver disease (8.05 vs. 5.1; *p* = 0.004), lower PT (Rho = −0.15; *p* = 0.029), higher bilirubin (Rho = 0.2; *p* = 0.004) and higher GGT (Rho = 0.21; *p* = 0.002).

Median CAP was 274 dB/m and 109/217 (50.3%) patients had CAP > 274 dB/m. CAP was significantly associated with higher BMI (Rho = 0.42; *p* < 0.001). No significant associations of CAP with other laboratory and clinical parameters presented in Table 1 were found (*p* > 0.05 for all analyses).

There was no significant correlation between LSM and CAP in the overall cohort of patients. However, in the subgroup of patients with chronic liver disease, a strong negative correlation between LSM and CAP (Rho = −0.81; *p* = 0.005) was observed. Regarding the patients with LSM > 7 kPa, 22/41 (53.7%) had CAP > 274 dB/m, indicative of the presence of liver steatosis.

LSM and CAP were not associated with the severity of clinal presentation of COVID19 as defined by 4CMS or need for HFNC oxygen supplementation (*p* > 0.05).

### 3.3. Correlations of FAST Score with Demographic, Biochemical and Clinical Parameters

Median FAST score was 0.31, with 119 (55.3%), 85 (39.5%) and 11 (5.1%) of patients having FAST values < 0.35, 0.35−0.67 and >0.67, respectively. FAST was expectedly associated with LSM (Rho = 0.36; *p* < 0.001), CAP (Rho = 0.4; *p* < 0.001) and AST (Rho = 0.81; *p* < 0.001), but also with higher values of ALT (Rho = 0.59; *p* < 0.001), GGT (Rho = 0.41; *p* < 0.001), CRP (Rho = 0.28; *p* < 0.001) and BMI (Rho = 0.21; *p* = 0.003). FAST score was not significantly associated with the severity of clinical presentation of COVID-19 as defined by 4CMS or HFNC oxygen supplementation (*p* > 0.05).

### 3.4. Relationships between LSM, CAP, FAST Score and 30-Day Clinical Outcomes

When analysed as a continuous variable, higher FAST score was significantly associated with the need for MV (median 0.4 vs. 0.29 in patients with and without MV use, *p* = 0.046) but no significant association with death or composite outcome of MV or death was present (*p* > 0.05 for both analyses). Neither LSM, nor CAP, evaluated as continuous variables, showed a significant association with adverse outcomes (*p* > 0.05 for all analyses).

**Figure 1:**
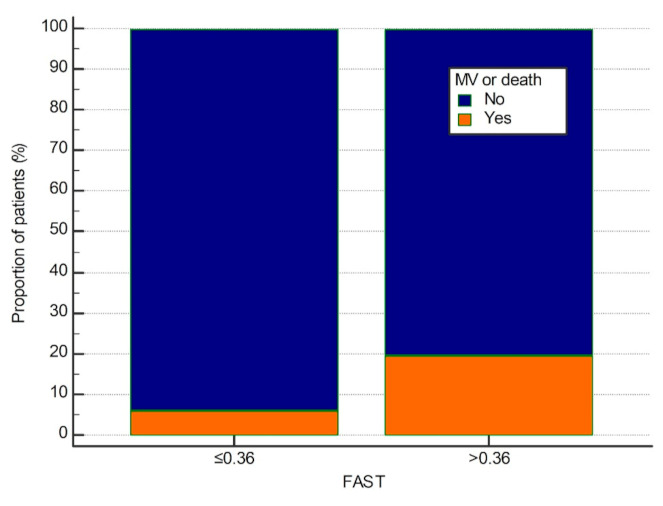
Thirty-day mechanical ventilation or death rates stratified according to the FAST > 0.36. MV, mechanical ventilation; FAST, FibroScan-AST score.

However, by using ROC curve analysis, we were able to establish optimal cut-offs for FAST of >0.36 (AUROC 0.632) for prediction of 30-day mortality or need of MV. Patients presenting with FAST > 0.36 (85/217 (39.2%)) experienced significantly higher risk of mechanical ventilation (OR = 4.39; *p* = 0.002), death (OR = 3.01; *p* = 0.018) and composite outcome of MV or death (OR = 3.81; *p* = 0.003). Thirty-day mechanical ventilation or death rates in patients with FAST > 0.36 and ≤0.36 were 19.8% and 6.2%, respectively (Figure 1). No similar cut-off level could be established for LSM and CAP.

We further investigated the relationship of FAST > 0.36 with reduced survival, need for MV and the composite outcome of MV or death in a series of multivariate logistic regression models. In the first set of models adjusted for age and sex, FAST > 0.36 remained significantly associated with higher occurrence of death (FAST > 0.36 OR 3.2, *p* = 0.021; age OR 1.07, *p* = 0.006; male sex *p* = 0.075), higher need for MV (FAST > 0.36 OR 4.49, *p* = 0.003; age OR 1.05, *p* = 0.024; male sex OR 3.98, *p* = 0.030) and higher occurrence of composite outcome (FAST > 0.36 OR 3.86, *p* = 0.005; age OR 1.06, *p* = 0.011; male sex OR 4.3, *p* = 0.021). In the second set of models presented in Table 2, after further adjusting for 4CMS and HFNC, FAST > 0.36 showed significant prognostic properties for predicting the need of MV and composite outcome of MV or death, independently of 4CMS and HFNC.

In addition, we evaluated how FAST > 0.36 would perform after adjusting for non-invasive biochemical scores (APRI, NSF, FIB-4). All three scores were associated with worse 30-day composite clinical outcome in univariate analyses (*p* < 0.05). However, inclusion of these scores in multivariate regression models did not change the relationships of FAST score, HFNC and 4CMS with clinical outcomes. Neither of the biochemical scores remained independently associated with the adverse composite outcome in this context. Furthermore, FAST score was analysed in the model adjusted for chronic metabolic comorbidities (obesity, diabetes, arterial hypertension, dyslipidaemia) and chronic liver disease, and again, only FAST > 0.36 remained significantly associated with the risk of composite adverse outcome (OR 3.68, 95%CI 1.47–9.21, *p* = 0.0054). Thus, FAST > 0.36 appears to have better prognostic properties compared to metabolic and chronic liver comorbidities in our cohort of patients.

## 4. Discussion

Our study demonstrates a lack of association between LSM and CAP as non-invasive surrogates for liver fibrosis and steatosis with the outcomes of COVID-19 defined as (a) severity of clinical presentation of COVID-19 and (b) 30-day mortality or need for MV. Nevertheless, higher FAST score, as a non-invasive surrogate for NASH with significant fibrosis, independently predicted the risk of composite outcome of MV or death.

FAST score was previously demonstrated as a reliable non-invasive tool to identify NASH patients with significant activity and liver fibrosis (19). We hypothesised that FAST score may correlate with more severe outcomes of COVID-19 given that patients with NASH are more likely to be obese with metabolic complications and the resulting liver inflammation may be accentuated by COVID 19 contributing to an adverse prognosis. Although we failed to observe a correlation between FAST score and clinical indicators of COVID-19 severity at admission to hospital, probably as the result of selection bias due to patients with critical illness at presentation being excluded from the study, we found FAST score > 0.36 was independently associated with the risk of MV and the composite adverse outcome of death or MV. However, without histological evidence, apparent linkage between FAST score as a non-invasive indicator of NASH and COVID-19 outcomes, as might be assumed based on current results, should be interpreted cautiously.

Previous investigations have clearly determined risk factors for the development of more severe COVID-19 disease and death, such as older age, male sex, increased blood pressure, presence of metabolic derangements such as diabetes and obesity, as well as some biochemical indicators (2–7). Obese patients are more likely to have fatty liver, which has been previously recognised as a risk factor for further deterioration of the metabolic profile of the affected individuals who are not COVID-19 patients [8,33]. Given the shared risk factors between NAFLD and severe COVID-19 disease, a more severe course of COVID-19 might hypothetically be expected amongst patients with fatty liver. Interestingly, in our cohort, FAST score, but not metabolic comorbidities, was independently associated with the risk of experiencing the composite adverse outcome at 30 days.

Liver involvement in COVID-19 has been proposed due to the high prevalence of elevated aminotransferases observed among the analysed cohorts of patients, but conflicting results were reported in terms of their origin, pathophysiological background, and impact on the course of the disease [12,13]. As transaminase elevations in COVID-19 may be multifactorial, the use of diagnostic tools for liver assessment which are not based on liver aminotransferases would be welcome. Whereas liver biopsy is obviously not acceptable for majority of typical cases of COVID-19, TE might represent reliable alternative. Transient elastography measures liver stiffness, as a surrogate of liver fibrosis which is also affected by liver inflammation, and CAP as the surrogate of liver steatosis [34]. Only a few studies have reported on the clinical utility of LSM and/or CAP in COVID-19 patients. Two studies (one European with 32 patients, and one Asian with 98 patients) reported a more severe clinical picture and higher mortality of COVID-19 patients with higher LSM. CAP was not associated with clinical outcomes, and both studies found correlation between LSM and liver transaminases [15,16]. The authors excluded patients with a history of chronic liver disease (CLD) and liver biopsy was not performed to support the findings obtained by TE. As opposed to these findings, a recent study from Barcelona failed to demonstrate any influence of LSM, CAP, baseline ALT and prior liver disease on the clinical course of COVID-19 in a cohort of 98 hospitalized patients, with 9% of them having CLD [17]. Nevertheless, elevated baseline AST especially in patients aged > 65 years was a strong predictor of adverse clinical outcomes. Recently, LSM and CAP were also investigated among patients with persisting post-acute COVID-19 syndrome and no history of liver disease [35]. LSM but not CAP was higher (but still within the normal range) in patients who suffered from a more severe form of COVID-19 during acute illness (5.08 kPa vs. 4.39 kPa, *p* = 0.017 for LSM, and 291.64 dB/m vs. 266.06 dB/m, *p* = 0.062 for CAP).

In agreement with data from the Campos-Varela study [21], our results demonstrate no association of LSM and CAP, when analysed individually, with the clinical severity and 30-day outcomes of COVID-19. Both LSM and CAP were not influenced by the levels of transaminases (up to 5× ULN as defined by inclusion criteria) nor were they correlated mutually. Consequently, LSM might not be a good individual predictor of clinical outcomes in a typical COVID-19 cohort with the low prevalence of chronic liver disease and normal to moderately elevated transaminases. Amongst the entire cohort of 217 patients analysed here, only 10 (4.6%) had a history of chronic liver disease, whereas a fourfold higher prevalence might have been assumed based on LSM. Indeed, 41/217 (18.9%) patients had LSM > 7 kPa and of them 12 (5.5%) had LSM ≥ 10 kPa, suggesting the presence of significant and advanced fibrosis, respectively. This could be due to previously unrecognized chronic liver disease amongst the patients coming from general population, now suffering from COVID-19, but alternatively could be secondary to overestimation of fibrosis stage by TE, as previously reported among patients with NAFLD where only 50% of patients with elevated LSM (≥9.6 kPa, suggestive of advanced fibrosis) had advanced fibrosis as confirmed by liver biopsy [36]. Due to high prevalence of overweight/obesity (almost 80%) and fatty liver (>50% with CAP > 274 dB/m), our cohort is comparable and might also follow this pattern of diagnostic performance of TE. Another reason for the increased proportion of patients with LSM > 7 kPa might be liver involvement in the inflammatory response to COVID-19 resulting in the increased liver stiffness.

We also analysed prognostic properties of biochemical non-invasive tests (APRI, FIB-4 and NFS) with respect to the clinical outcomes of patients with COVID-19. Although significantly different values between the patients with different outcomes could be demonstrated for each test, in multivariate regression analysis they failed to independently predict the risk of MV or death. Non-invasive biochemical tests potentially suffer from limitations in assessing liver health in the setting of COVID-19. In particular, they were invented as the indirect indicators of liver fibrosis in patients with chronic liver disease, and consist of liver aminotransferases, platelets and certain demographic indicators. In the setting of COVID-19, liver aminotransferases are elevated in up to 75% of patients without a history of chronic liver disease, and platelets might be decreased due to direct viral effect, immunological mechanisms or induced by medications such as heparin. Therefore, the use of biochemical non-invasive tests in this setting might not be reliable.

Our study has some limitations: liver biopsy is insufficient to reveal correlation between elastographic measurements and histological changes; TE was performed by several operators and we were not able to assess the interobserver variability of LSM and CAP measurements. On the other hand, this is the largest study thus far to report the performance of LSM and CAP by TE, with regard to their correlation with clinical severity of COVID-19 and 30-day outcome, and is the first to evaluate the FAST score in this regard. A strength of our study is that all patients underwent standardized diagnostic and treatment protocols and our cohort is representative of a typical hospitalised COVID-19 patient cohort with a low prevalence of chronic liver disease.

In conclusion, these data demonstrate that LSM and CAP as non-invasive surrogates for liver fibrosis and steatosis do not correlate with the severity and clinical outcomes of COVID-19 in a typical cohort of hospitalised patients with low prevalence of chronic liver disease and normal or moderately elevated transaminases. FAST score > 0.36 was for the first time demonstrated to independently predict the risk of composite 30-day adverse outcomes. Even after adjustment for the presence of chronic metabolic comorbidities and noninvasive biochemical fibrosis indices, FAST score remained significantly associated with the risk of 30-day composite adverse outcome of mechanical ventilation or death. However, the issue of liver involvement in COVID-19 might not be precisely addressed based on the available results until more histological data are collected.

## Figures and Tables

**Table 1:** Patient characteristics in overall cohort and stratified according to the composite outcome of mechanical ventilation or 30-day mortality.

	Overall	MV or Death No	MV or Death Yes	*p*-Value
Total number	217	192	25	-
Age (years)	65 IQR (55–70)	64 IQR (55–70)	70 IQR (62–75)	**0.014**
Sex Male Female	144/217 (66.4%) 73/217 (33.6%)	123/192 (64.1%) 69/192 (35.9%)	21/25 (84%) 4/25 (16%)	**0.047**
BMI (kg/m^2^)	28.3 IQR (25.4–31.5)	28.4 IQR (25.4–31.6)	26.9 IQR (25.65–30.9)	0.342
Probe type M XL	140/217 (64.5%) 77/217 (35.5%)	121/192 (63%) 71/192 (37%)	19/25 (76%) 6/25 (24%)	0.203
SCD (mm)	21 IQR (19–25)	22 IQR (18–25.25)	20 IQR (19–23)	0.652
Chronic liver disease	10/217 (4.6%)	8/192 (4.2%)	2/25 (8%)	0.323
LSM (kPa)	5.2 IQR (4.1–6.5)	5.1 IQR (4.18–6.53)	5.3 IQR (4.1–6.3)	0.873
LSM IQR (%)	13 IQR (9–20)	13 IQR (9–20)	13 IQR (8–18)	0.419
CAP (dB/m)	274 IQR (232–321)	273 IQR (233.5–322)	284 IQR (228–301)	0.643
CAP IQR (dB/m)	31 IQR (22–43)	31 IQR (22–43)	31 IQR (20–38)	0.487
FAST score	0.31 IQR (0.16–0.45)	0.3 IQR (0.14–0.45)	0.4 IQR (0.25–0.47)	0.112
WBC (×10^9^/L)	7.8 IQR (5.45–11.1)	8 IQR (5.53–11.1)	6.9 IQR (3.8–10.9)	0.545
RBC (×10^12^/L)	4.5 IQR (4.14–4.89)	4.5 IQR (4.14–4.88)	4.5 IQR (4.12–4.9)	0.872
Platelets (×10^9^/L)	237 IQR (173–327.5)	243.5 IQR (178.25–334)	204 IQR (144–264)	0.087
PT (Quick, %)	101 IQR (90–108)	101.5 IQR (92–108)	89 IQR (76–107)	0.059
Bilirubin (umol/L)	10.6 IQR (8.6–15.4)	10.6 IQR (8.6–15.45)	10.7 IQR (8.1–15.2)	0.979
AST (IU/L)	39 IQR (27–61)	38 IQR (26–57)	58 IQR (35–84)	0.014
ALT (IU/L)	38 IQR (24–63)	37.5 IQR (23.25–62)	41 IQR (27–67)	0.614
ALP (IU/L)	62 IQR (51–78.5)	61 IQR (50–77)	64 IQR (58–86)	0.166
GGT (IU/L)	42 IQR (25.5–82)	43 IQR (27–85.75)	37 IQR (24–56)	0.165
CRP (mg/L)	71.8 IQR (31.2–128.3	69.5 IQR (28.03–122.23)	100.4 IQR (48.8–138.2)	0.072
Albumin (g/L)	32.5 IQR (30–35)	33 IQR (30–35)	32 IQR (30–34)	0.433
4C mortality score	7 IQR (5–9)	7 IQR (4.5–9)	10 IQR (8.75–11)	**<0.001**
HFNC oxygenation (N, %)	24/217 (11.1%)	14/192 (7.3%)	10/25 (40%)	**<0.001**

Table legend: BMI, body mass index; CAP, controlled attenuation parameter; FAST, FibroScan-AST score; LSM, liver stiffness measurement; IQR, interquartile range; SCD, skin-to-liver capsule distance; CRP, C-reactive protein; HFNC, high-flow nasal cannula; MV, mechanical ventilation; ALT, alanine transaminase; AST, aspartate aminotransferase; ALP, alkaline phosphatase; GGT, gamma glutamyl transferase; PT, prothrombin time; RBC, red blood cells; WBC, white blood cells.

**Table 2:** Multivariate logistic regression models adjusted for age, sex and COVID-19 severity (4C mortality score, use of HFNC oxygenation) investigating association of FAST score with 30-day mortality, mechanical ventilation and mechanical ventilation or death.

	30-Day Mortality *p*-Value and OR with 95% C.I.	Mechanical Ventilation *p*-Value and OR with 95% C.I.	Mechanical Ventilation or Death *p*-Value and OR with 95% C.I.
FAST > 0.36	*p* = 0.285 OR = 1.79 (0.61–5.23)	*p* = 0.019 * OR = 3.78 (1.24–11.5)	*p* = 0.036 * OR = 3.11 (1.08–8.97)
Age (years)	*p* = 0.321 OR = 0.95 (0.88–1.04)	*p* = 0.136 OR = 0.94 (0.87–1.02)	*p* = 0.199 OR = 0.95 (0.88–1.03)
Male sex	*p* = 0.761 OR = 0.83 (0.24–2.81)	*p* = 0.660 OR = 1.33 (0.36–4.92)	*p* = 0.542 OR = 1.49 (0.41–5.33)
4C mortality score	*p* = 0.004 * OR = 1.83 (1.31–2.57)	*p* = 0.001 * OR = 1.72 (1.25–2.38)	*p* = 0.001 * OR = 1.71 (1.24–2.35)
HFNC oxygenation	*p* = 0.002 * OR = 7.4 (2.15–24.44)	*p* < 0.001 * OR = 7.76 (2.4–25.08)	*p* < 0.001 * OR = 7.17 (2.24–22.92)

Table legend: BMI, body mass index; FAST, FibroScan-AST score; LSM, liver stiffness measurement; IQR, interquartile range; HFNC, high-flow nasal cannula. * Statistically significant at level *p* < 0.05.

## Data Availability

The data presented in this study are available from the corresponding author upon reasonable request.

## Abbreviations

ACE2	Angiotensin-converting enzyme II
ALP	Alkaline phosphatase
ALT	Alanine aminotransferase
APRI	AST to platelet ratio index
AST	Aspartate aminotransferase
BMI	Body mass index
CAP	Controlled attenuation parameter
COVID-19	Coronavirus disease 2019
CRP	C-reactive protein
FAST	FibroScan-AST score
FIB-4	Fibrosis 4 index
GGT	Gamma glutamyl transferase
HFNC	High flow nasal cannula
IQR	Interquartile range
LSM	Liver stiffness measurement
MV	Mechanical ventilation
NAFLD	Non-alcoholic fatty liver disease
NASH	Non-alcoholic steatohepatitis
NFS	Non-alcoholic fatty liver disease fibrosis score
PT	Prothrombin time
RBC	Red blood cells
SCD	Skin-to-liver capsule distance
WBC	White blood cells
